# Dataset from chemical gas sensor array in turbulent wind tunnel

**DOI:** 10.1016/j.dib.2015.02.014

**Published:** 2015-03-04

**Authors:** Jordi Fonollosa, Irene Rodríguez-Luján, Marco Trincavelli, Ramón Huerta

**Affiliations:** BioCircuits Institute, University of California, San Diego, La Jolla, CA 92093, USA

**Keywords:** Chemometrics, Machine olfaction, Electronic nose, Chemical sensing, Machine learning, Open Sampling System

## Abstract

The dataset includes the acquired time series of a chemical detection platform exposed to different gas conditions in a turbulent wind tunnel. The chemo-sensory elements were sampling directly the environment. In contrast to traditional approaches that include measurement chambers, open sampling systems are sensitive to dispersion mechanisms of gaseous chemical analytes, namely diffusion, turbulence, and advection, making the identification and monitoring of chemical substances more challenging.

The sensing platform included 72 metal-oxide gas sensors that were positioned at 6 different locations of the wind tunnel. At each location*,* 10 distinct chemical gases were released in the wind tunnel*,* the sensors were evaluated at 5 different operating temperatures*,* and 3 different wind speeds were generated in the wind tunnel to induce different levels of turbulence. Moreover*,* each configuration was repeated 20 times*,* yielding a dataset of 18,000 measurements. The dataset was collected over a period of 16 months.

The data is related to “On the performance of gas sensor arrays in open sampling systems using Inhibitory Support Vector Machines”, by Vergara et al.[1].

The dataset can be accessed publicly at the UCI repository upon citation of [1]: http://archive.ics.uci.edu/ml/datasets/Gas+sensor+arrays+in+open+sampling+settings

**Specifications Table**

**Table:** 

Subject area	Chemistry
More specific subject area	Chemometrics, Machine Olfaction, Electronic Nose, Chemical Sensing, Machine Learning
Type of data	Text Files
How data was acquired	Metal Oxide (MOX) gas sensors provided by Figaro Inc. placed in a turbulent wind tunnel. Temperature and RH were recorded continuously with SHT15 sensor (Sensirion).
Data format	Raw data. Time-series.
Experimental factors	For each measurement 72 time series corresponding to MOX sensors׳ conductivity are provided. Temperature and humidity are provided in additional time series.
Experimental features	Sensors were exposed to clean air before and after sample presentation to acquire rising/decay transient portions of the signals.
Data source location	San Diego, California, US.
Data accessibility	Data in public repository:
	http://archive.ics.uci.edu/ml/datasets/Gas+sensor+arrays+in+open+sampling+settings Citation of [1] is required.

## Value of the data

• Extensive dataset (18,000 measurements) generated from chemical sensors exposed to ten different volatiles, at different locations, and five operating temperatures.
• Realistic scenario: sensors sampling in turbulent environment, with different levels of turbulence.
• Response of the same chemical detection platform measured consistently over a period of 16 months.
• Complete time series are provided, including baseline, rising/decay portion, and steady state. System sensitive to gas turbulence.
• Dataset suitable for the benchmark of different Machine Learning techniques for chemical sensing.

## 1 Experimental design, materials and methods

### 1.1 Experimental setup

#### 1.1.1 Chemical detection platform

Conductometric sensing principles have been widely studied in several types of gas sensing schemes because they are stable in many environments and within a wide temperature range, sensitive to many analytes at a wide variety of concentrations, respond quickly and reversibly, and are inexpensive, while performing reasonably well in discriminating chemical analytes [2]. Although they have been predominantly used in isolated settings that include measurement chambers, their high sensitivity and rapid response to a wide variety of volatiles distinguishes MOX sensors as suitable chemo-transducers for ambient conditions.

We designed a general purpose chemical sensing platform containing nine portable chemo-sensory modules, each endowed with eight commercialized metal oxide gas sensors, provided by Figaro Inc., to detect analytes and follow the changes of their concentration in a wind tunnel facility. The sensor׳s response magnitude to the chemical analyte is signaled by a change in the electrical conductivity of the sensor׳s film, which is tightly correlated with the analyte concentration present on its surface. Hence, changes in the analyte concentration (mostly due to patches and eddies in the chemical plume) are reflected in the sensor׳s response in real-time and are the origin of the temporal resolution (i.e. fluctuations in the time series).

The active surface chemistry (both sensing layer material and sensor׳s operating temperature) is a decisive factor in the sensitivity and the selectivity of the sensing elements. In particular, the sensing layers used in each of our sensory modules represent six different sensitive surfaces, as listed in Table 1. Sensitivity to chemicals and nominal resistance may change significantly among MOX gas sensors, even for sensors of the same type [3]. Hence, we included some replicas of the same sensor type in the arrays, enabling further studies on sensors׳ reproducibility and development of algorithms to alleviate sensors׳ variability. On the other hand, the operating temperature of the sensors in our array is adjustable by applying a voltage to the built-in heater of each sensor. The sensors׳ operating temperature affects all aspects of the sensor response, including selectivity, sensitivity and response time of the sensor to volatiles [4].

In our particular chemical sensing platform, each portable chemosensory module is integrated with a customized sensor controller implemented with a microprocessor MSP430F247 (Texas Instruments Inc.). This controller enables continuous data collection from the eight chemical sensors through a 12-bit resolution analog-to-digital converter (ADC) at a sampling rate of 100 Hz, the control of the sensor heater temperature by means of 10 ms period and 6 V amplitude Pulse-Width-Modulated driving signals, and the two-way communication with a computer to acquire sensors׳ signals and control sensors׳ heaters. In particular, we set the operating temperature of the sensors at 5 different levels, controlled by the voltage applied in the heater: from 4 V to 6 V with an resolution of 0.5 V.

#### 1.1.2 Wind tunnel

**Fig. 1:**
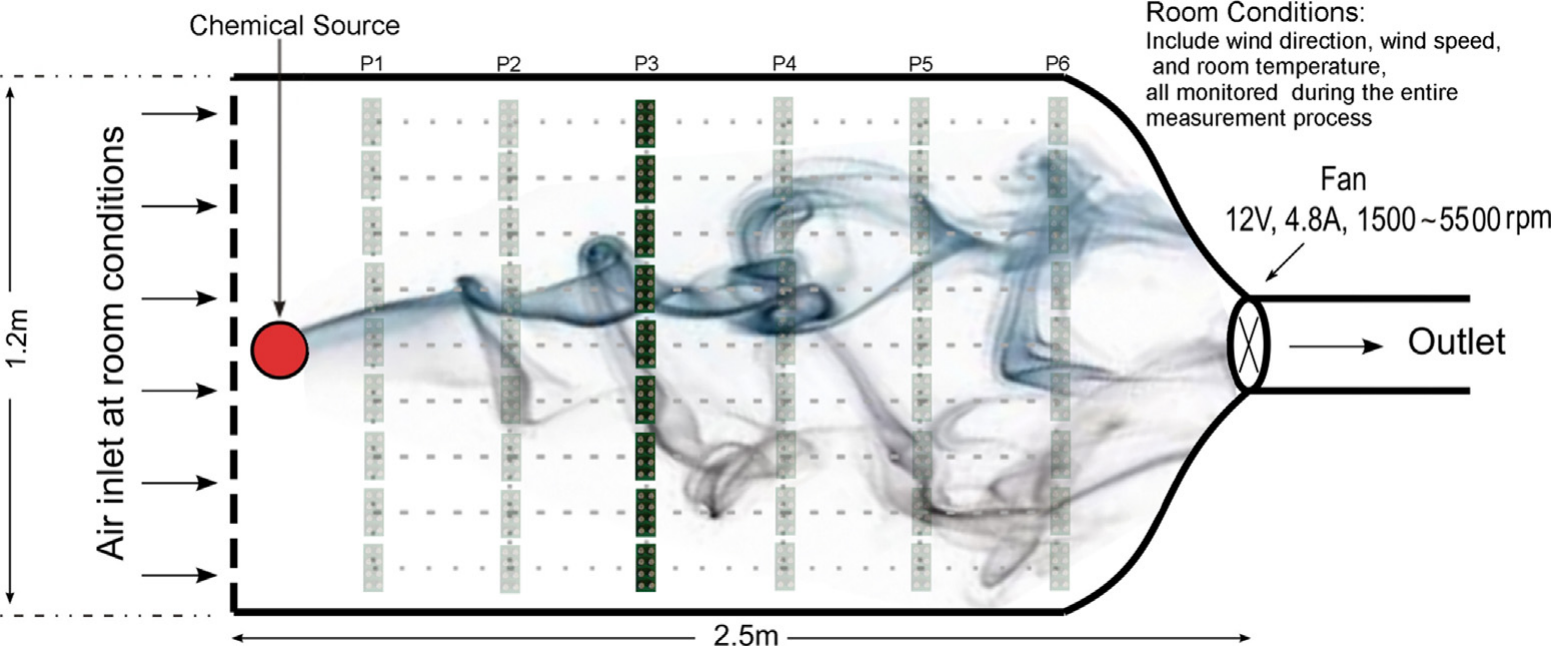
Wind tunnel used to collect time series data from sensor arrays. The displacements of the 6 lines are labeled in the schema as P1-P6.

We constructed a 2.5 m×1.2 m×0.4 m wind tunnel (see Fig. 1), a research test-bed facility endowed with a computer-supervised mass flow controller system. The resulting wind tunnel operates in a propulsion open-cycle mode, by continuously drawing external turbulent air throughout the tunnel and exhausting it back to the outside, thereby creating a relatively less-turbulent airflow moving downstream towards the end of the test field. This operational mode is particularly crucial for applications that require injecting chemical poisonous agents or explosive mixtures because it prevents saturation. The gas source in the wind tunnel was controlled by a set of mass flow controllers that, along with calibrated pressurized gas cylinders provided by Airgas Inc., provided the chemical substances of interest at selected concentrations. To create various distinct artificial airflows in the wind tunnel, we utilize a multiple-step motor-driven exhaust fan located inside the wind tunnel at the outlet of the test section rotating at three different constant rotational speeds: 1500 rpm (25 Hz), 3900 rpm (65 Hz), 5500 rpm (91.66 Hz). We estimated the induced wind speed by means of two anemometers (Gill Windsonic). The mean wind speed in the axis of the wind tunnel increased with the rotational speed: 0.1 m/s, 0.21 m/s, and 0.34 m/s respectively.

The wind tunnel was used to collect time series from sensor arrays placed at different locations. The nine detection units were placed in six lines normal to the wind flow. Each line included 9 landmarks evenly distributed along the line to complete a grid of 54 evaluation landmarks. Each of the nine detection units was always placed on the same location of each sensing line, i.e. each unit was always placed at the same distance with respect to the axis of the gas plume. Finally, to measure the ambient temperature and humidity during the entire experiment in the wind tunnel we utilized the sensor SHT15 (Sensirion).

## 2 Methods

We compiled a very extensive dataset utilizing nine portable sensor array modules – each endowed with eight metal oxide gas sensors – positioned at six different line locations normal to the wind direction, creating thereby a total number of 54 measurement locations. In particular, our dataset consists of 10 chemical analyte species. Table 2 shows the entire list of chemical analyte as well as their nominal concentration values at the outlet of the gas source.

**Fig. 2:**
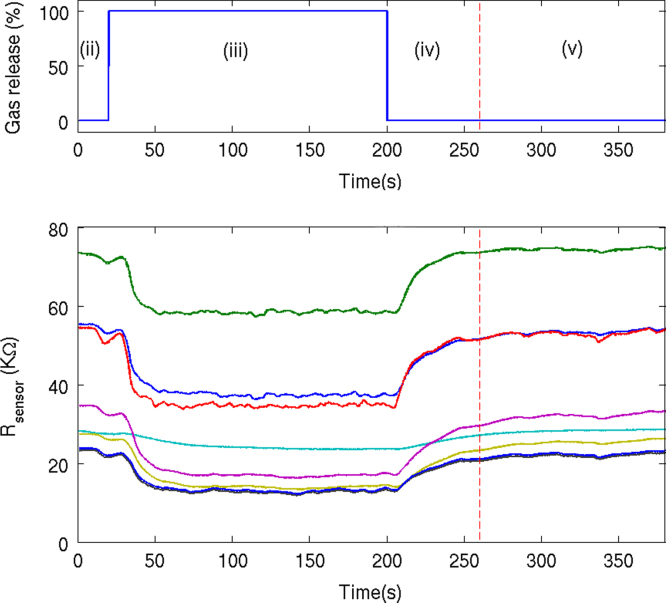
Multivariate response of a 8-sensor array when methane is released in the wind tunnel. The sensors’ responses are affected by the air turbulence present in the wind tunnel, inducing fluctuations in the acquired signals. The experimental protocol carried out to acquire the signals of the sensors under different conditions consists of (i) Set the operating temperature and location of the sensors and the wind speed of the fans. (ii) During 20 s, measure the baseline of the sensors׳ signal while no chemical compound is released. (iii) Release the chemical compound during 3 min. (iv) One minute circulating clean air to acquire the sensors׳ recovery signals. (v) Two additional minutes purging at maximum wind speed to clean the wind tunnel. Each measurement is considered to finish in point (iv) since the sensors׳ signals are not recorded during the cleaning phase (v).

To construct the dataset, we adopted the following procedure. First, we positioned our chemo-sensory platform, ie the 9 sensing units, in one of the six fixed line positions indicated in the wind tunnel, and set the chemical sensors to one of the predefined surface operating temperatures. One of the predefined airflows was then individually induced into the wind tunnel by the exhaust fan, generating thereby the turbulent airflow within the test section of the wind tunnel. This stage constituted a preliminary phase that allowed to reach a quasi-stationary situation and to measure the baseline of the sensor responses for 20 s before the chemical analyte was released. We then randomly selected one of the ten described chemical volatiles and released it into the tunnel at the source for three minutes. The chemical analyte circulated throughout the wind tunnel while recording the generated sensor time series. Note that the concentration reported in Table 2 represents only the concentration at the outlet of the gas source. Concentration disperses as the generated gas plume spreads out along the wind tunnel. After that step, the chemical analyte was removed and the test section was ventilated utilizing clean air circulating through the sampling setting at the same wind speed for another minute. Fig. 2 shows the typical response of the sensors after a complete measurement was recorded.

This measurement procedure was reproduced exactly for each gas category exposure, landmark location in the wind tunnel, operating temperature, airflow velocity, and repetitions in a random order up until all combinations were covered. The resulting dataset in the end comprises 18,000 72-dimensional time recordings. Hence, the total number of measurements is distributed as follows: 3 different wind speeds, 5 different sensors׳ temperatures, 10 gases, 6 locations in the wind tunnel, and 20 replicas.

Finally, note that although different induced wind speeds strongly influence the structure and spatial distribution of the generated gas plumes – in the sense that slow fan speeds induce less stable patterns of the air flow direction, resulting in wider gas plumes, whereas faster velocities in the wind generate narrower gas plumes – there is no symmetry in the spatial distribution of the plume with respect to the main axis (i.e., the line connecting the chemical analyte source to the exhaust). A plume demonstrating a perfect symmetry in real environmental conditions is rare due to the existent non-symmetry of the volume enclosing the field, the inhomogeneous temperature in the ambient, and the variability of the flow direction.

## Figures and Tables

**Table 1:** MOX sensors included in the 8-sensor array. The manufacturer adapts the sensing layer to detect different target gases.

Sensor type	Number of units in each module
TGS2611	1
TGS2612	1
TGS2610	1
TGS2600	1
TGS2602	2
TGS2620	2

**Table 2:** Gases and corresponding concentrations at the outlet of the gas source. Note that the actual concentration in the wind tunnel decreases as the generated gas plume spreads out along the tunnel. All the chemicals are released at a constant flow of 320 sccm.

Analyte	Concentration at the gas source (ppm)
acetone	2500
acetaldehyde	500
ammonia	10,000
butanol (butyl-alcohol)	100
ethylene	500
methane	1000
methanol	200
carbon monoxide	1000/4000
benzene	200
toluene	200
